# Looks Like Neurosyphilis, Feels Like Guillain-Barre: At the Confluence of Infection and Immunology

**DOI:** 10.7759/cureus.26318

**Published:** 2022-06-25

**Authors:** Joseph I Berger, Kasun Vernon, Farid Abdo, Sandeep Gulati, Radhika Hariharan

**Affiliations:** 1 Internal Medicine, St. John's Riverside Hospital, Yonkers, USA; 2 Radiology, St. John's Riverside Hospital, Yonkers, USA; 3 Neurology, St. John's Riverside Hospital, Yonkers, USA; 4 Infectious Disease, St. John's Riverside Hospital, Yonkers, USA

**Keywords:** guillain-barre syndrome, csf pleocytosis, vaccine, covid-19, emg, rpr, vdrl, csf, neurosyphilis, syphilis

## Abstract

We present a 51-year-old male, with a past medical history of type 2 insulin-dependent diabetes mellitus (T2IDDM) without neuropathy, coronavirus disease 2019 (COVID-19) in April 2020 without residual symptoms, Raynaud's, and recent occupational outdoor exposure to insects as a construction manager who came to the emergency room complaining of a three-week history of bilateral progressive numbness and weakness beginning in his lower extremities and ascending toward his pelvis. Notably, he received the second dose of his Moderna COVID-19 vaccine one week prior to symptom onset and four weeks prior to admission. He also reported a recent appearance of a maculopapular rash on his upper extremities and flanks. Physical exam was remarkable for bilateral distal motor weakness in the upper and lower extremities with associated paresthesia and decreased reflexes in the lower extremities. The patient had slight ataxia and difficulty with heel walk and toe walk. Notably, the cranial nerve exam was normal, and the patient was afebrile. Intravenous immune globulin (IVIG) was started empirically for the treatment of Guillain-Barre syndrome (GBS), and doxycycline 100mg intravenous twice a day and ceftriaxone 2g intravenous daily were started for possible tick-borne disease. Subsequently, rapid plasma reagin (RPR) returned reactive at 1:64, and cerebral spinal fluid (CSF) venereal disease research laboratory (VDRL) test was reactive at 1:2 with markedly elevated protein and pleocytosis. Human immunodeficiency virus (HIV) testing was negative. Lyme disease testing was negative. Nerve conduction studies (NCS) and electromyography (EMG) showed a sensorimotor polyneuropathy with mixed demyelinating and axonal features. IVIG was continued for a total of five days, and antibiotics were changed to penicillin G (PCN G) for a total of 14 days for definitive treatment of early neurosyphilis (NS). While both clinical and laboratory findings confirm a positive diagnosis of NS, the patient’s CSF composition showed very elevated total protein levels and pleocytosis. Additionally, his early peripheral neuropathy and EMG findings are not characteristics of a single disease and, instead, suggested a mixed pathology. We postulate that this patient had confirmed secondary syphilis with early NS associated with, and possibly correlated with, a simultaneous episode of acute inflammatory demyelinating polyneuropathy (AIDP) and/or a vaccine-related phenomenon.

## Introduction

Syphilis is a sexually transmitted infection caused by the spirochete *Treponema pallidum* and is referred to as the “great pretender” due to the wide array of symptoms at the time of diagnosis. As a result, a syphilis workup is often undertaken during an early investigation of pathologies involving neurological or dermatological findings when a clear diagnosis is not present. However, it is important to note that a diagnosis of syphilis does not rule out the existence of another concomitant disease, especially in the presence of convincing evidence [1,2]. Our patient presented with history, signs, and symptoms concerning for several etiologies including infectious, immune mediated, and vaccine related. Additionally, our patient's past medical history of type 2 insulin-dependent diabetes mellitus (T2IDDM) without neuropathy, coronavirus disease 2019 (COVID-19) without residual symptoms, and recent COVID-19 vaccination served as confounding variables.

Our review of the literature has revealed 10 specific reports of syphilis associated with Guillain-Barre syndrome (GBS) type symptoms. Each of these cases demonstrated signs of peripheral neuropathy or weakness in patients who had syphilis infections, not likely to have progressed to tabes dorsalis where such neuropathy would be present. Additionally, in these 10 cases, only four were treated with penicillin (PCN) alone, with full or almost complete recovery in all cases. Three cases were treated with PCN and intravenous immunoglobulin (IVIG) with two patients showing improvement and one lost to follow-up. One case was treated with PCN and plasmapheresis with initial improvement; however, the patient was also lost to follow-up. The remaining two cases were treated with fever therapy, and both showed moderate recovery [3-11]. Additionally, recent literature has shown GBS associated with coronavirus disease 2019 (COVID-19) vaccinations from multiple pharmaceutical companies [12]. We thus posit that infection with syphilis, as well as recent COVID-19 vaccination, may have contributed to our patient’s concomitant GBS-like symptoms. This article was previously presented as an abstract and poster presentation at the New York Chapter American College of Physicians Annual Scientific Meeting E-Poster Presentations on Thursday, September 23, 2021, by the submitting author of this report.

## Case presentation

**Figure 1:**
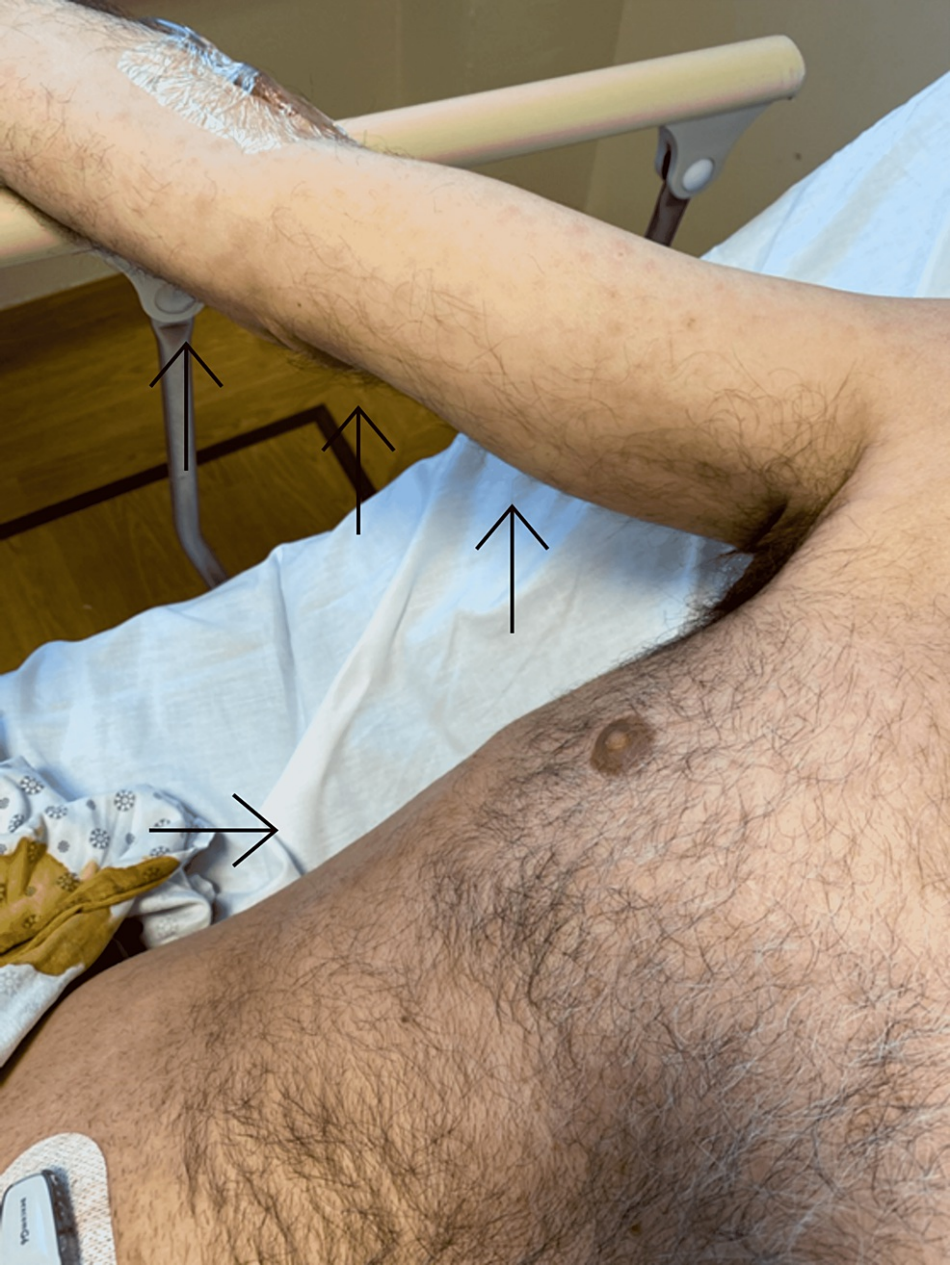
Maculopapular rash demonstrated on the right upper extremity and flank, designated by black arrows.

We present a 51-year-old male, with a past medical history of type 2 insulin-dependent diabetes mellitus (T2IDDM) without neuropathy, coronavirus disease 2019 (COVID-19) in April 2020 without residual symptoms, Raynaud's, and recent occupational outdoor exposure to insects as a construction manager who came to the emergency room complaining of a three-week history of bilateral progressive numbness and weakness beginning in his lower extremities and ascending toward his pelvis. He also reported the recent appearance of a maculopapular rash on his upper extremities and flanks (Figure 1). The patient is monogamous with his wife and does not use barrier protection. The patient denied any extramarital contacts. Sexual history from his wife was unavailable. He denied any previous history of syphilis or treatment for syphilis. Additionally, he denied any recent personality changes or ocular complaints.

The patient received his second Moderna COVID-19 vaccine one week prior to the appearance of symptoms and four weeks prior to admission. Prior to his admission, he was prescribed steroids and muscle relaxants without effect. Additionally, the patient endorsed recent outpatient lumbar magnetic resonance imaging (MRI) which was negative for acute pathology. Our physical exam was remarkable for distal motor weakness 4+/5 in both the upper and lower extremities with associated paresthesia and 2+ reflexes in the upper extremities with only limited trace Achilles reflex in the lower extremities. Notably, the cranial nerve exam was normal, and the patient was afebrile. Additionally, on gait examination, the patient had slight ataxia and difficulty with heel walk and toe walk. The clinical findings of ascending weakness after vaccination prompted an initial clinical diagnosis of GBS, and the patient was transferred to the intensive care unit (ICU) for respiratory monitoring.

**Figure 2:**
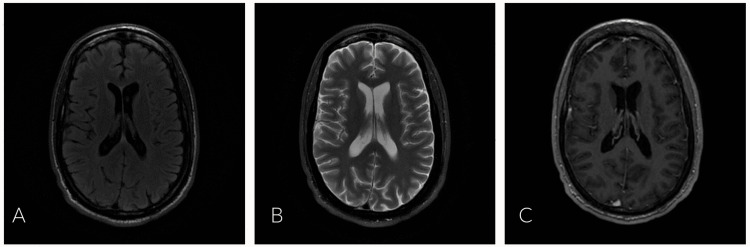
MRI brain: axial images with and without contrast A: MRI brain, axial view, T2 flair. B: MRI brain, axial view, T2 with fat suppression. C: MRI brain, axial view, three-dimensional fast spoiled gradient-echo with contrast. All images demonstrate no evidence of enhancing intra-axial or extra-axial neoplasm, demyelinating plaques, hemorrhage or acute infarction, hydrocephalus, or suspicious meningeal enhancement. Permission to reproduce obtained from the St. John's Riverside Hospital Department of Radiology and reviewed by co-author, Dr. Farid Abdo.

**Figure 3:**
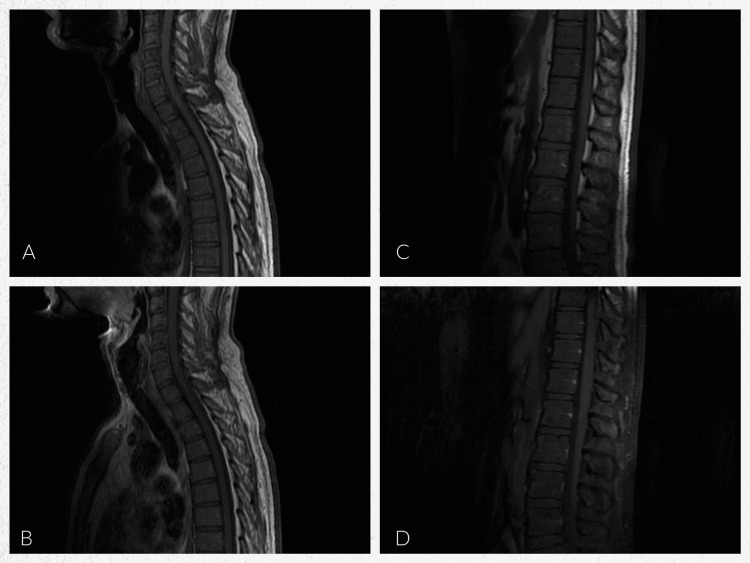
MRI cervical, thoracic, and lumbar spine with and without contrast A: MRI cervical and thoracic spine, sagittal view, T1 without contrast. B: MRI cervical and thoracic spine, sagittal view, T1 contrast enhanced. C: MRI thoracic spine and lumbar spine, sagittal view, T1 without contrast. D: MRI thoracic spine and lumbar spine, sagittal view, T1 fat-suppressed contrast enhanced. Imaging of the cervical spine showed no suspicious pathologic bone marrow replacement in the cervical vertebrae and no evidence of acute compression fracture, paraspinal mass, or enhancing intramedullary or epidural mass lesions. No evidence of a syrinx. Imaging of the thoracic spine showed no evidence of thoracic cord enlargement or suspicious enhancing intramedullary or epidural mass lesion and no evidence of midline or foraminal herniation or cord compression. Imaging of the lumbar spine showed no evidence of suspicious signal intensity changes within the cauda equina, no evidence of pathologic bone marrow replacement in the lumbar vertebrae, no acute compression fracture, and no paraspinal mass. There was no evidence of enhancing intramedullary or epidural mass lesions or spinal stenosis. Permission to reproduce obtained from the St. John's Riverside Hospital Department of Radiology and reviewed by co-author, Dr. Farid Abdo.

Rapid plasma reagin (RPR) and human immunodeficiency virus (HIV) serology were ordered as part of the evaluation of the maculopapular rash. Infectious Disease, Neurology and Physical Medicine and Rehabilitation were consulted for their expertise. Lumbar puncture (LP), nerve conduction studies (NCS), electromyography (EMG), and MRI of the brain and spine were obtained to aid in diagnosis (Figures 2A-2C, 3A-3D). Empiric treatment with IVIG was started for presumed GBS, and doxycycline 100mg intravenous twice a day and ceftriaxone 2g intravenous daily were started for empiric Lyme disease coverage. The patient demonstrated improvement with decreased numbness and improved gait by day 3. MRI brain/spine showed no acute change or evidence of spinal cord myelitis. RPR was reactive at 1:64, HIV was negative, and serum Lyme disease was negative. Cerebral spinal fluid (CSF) findings were notable for a reactive venereal disease research laboratory (VDRL), with pleocytosis and elevated total protein. CSF Lyme was negative. Antinuclear antibody and double-stranded deoxyribonucleic acid (DS-DNA) were negative. Screening for antibodies against gangliosides was not performed. All reference ranges are from our performing laboratory (Table 1). Sensory nerve conduction studies showed no response at the median nerves bilaterally, as well as bilateral slowed conduction velocity at the ulnar nerve. Sural sparing of the right lower extremity was present; the left lower extremity was not tested. Motor NCS showed no response in the left median nerve and decreased velocity in the left ulnar nerve; the right upper extremity was not tested. Findings on EMG were significant for reduced recruitment along with evidence of acute denervation. Electrodiagnostic interpretation demonstrated a sensorimotor polyneuropathy with mixed demyelinating and axonal features. 

**Table 1:** Laboratory values demonstrating serum serology and cerebral spinal fluid composition HIV: human immunodeficiency virus, RNA: ribonucleic acid, SARS-CoV-2: severe acute respiratory syndrome coronavirus 2. All values and reference ranges are provided by our in-hospital laboratory.

Laboratory values		
Serum serology	Values	Normal values
Rapid plasma reagin	Reactive 1:64	Nonreactive
Lyme disease	<0.91 (negative)	0.00-0.91
QuantiFERON	Negative	Negative
HIV	Negative	Negative
SARS-CoV-2 RNA	Negative	Negative
Antinuclear antibody (ANA)	Negative	<1:80
Antidouble-stranded deoxyribonucleic acid antibody (DS-DNA)	2	<5
Cerebral spinal fluid	Values	Normal values
Appearance	Hazy	Clear
Color	Colorless	Colorless
White blood cells (cells/mm^3^)	73	0-5
Lymphocytes (percentage/cells)	86%	40%-80%
Neutrophils (percentage/cells)	9%	0%-6%
Red blood cells (cells/mm^3^)	9	0
Total protein (mg/dL)	148	15-45
Glucose (mg/dL)	120	40-70
Venereal disease research laboratory	Reactive 1:2	Nonreactive
Lyme disease DNA	Negative	Negative

CSF leukocytosis in conjunction with the serum RPR and positive CSF VDRL confirmed the diagnosis of neurosyphilis (NS). Ceftriaxone was discontinued, and IV penicillin G (PCN G) was started as a definitive treatment of NS as the patient had no medication allergies, and IVIG was continued [1]. The patient’s physical and neurologic symptoms continued to improve, and he was discharged from the ICU after receiving five doses of IVIG and treatment with IV PCN G to be continued outpatient for a total of 14 days high-dose IV PCN G. Outpatient follow-up one month after treatment completion revealed that the patient’s weakness and sensory loss had markedly improved. Additionally, repeat nerve conduction studies did not show any clear evidence of demyelination. The patient did not follow up for his three-month repeat RPR.

## Discussion

The diagnosis of NS is multifaceted, and there is no current gold standard; however, it can be classified as confirmed or presumptive NS. A confirmed diagnosis is demonstrated by a reactive VDRL in the CSF. A presumptive diagnosis of NS is demonstrated by a negative CSF VDRL with increased CSF protein >45 mg/dL or an elevated CSF white blood cell (WBC) count >5 cells/mm^3^, alongside a reactive serum syphilis test, in combination with clinical symptoms resembling NS with no alternate cause [13]. In a 2015 study done by Merins and Hahn, the average CSF protein present in non-HIV patients with confirmed NS was 57.7 mg/dL [14]. Early forms of NS are subcategorized into asymptomatic NS and symptomatic; late forms are classified into general paresis and tabes dorsalis. The early forms typically affect the CSF, meninges, and vasculature, while the late forms affect the brain and spinal cord parenchyma. Early forms can occur within the first few weeks to months after infection, while late forms may present decades after the initial infection [15]. Early symptoms include headache, nausea, vomiting, neck stiffness, photophobia, cranial nerve deficits, spastic weakness, sensory loss, and muscular atrophy, while late symptoms include diminished reflexes, vibratory, and proprioceptive impairments [16].

Guillain-Barre syndrome is thought to result from an immune response to a preceding infection or immunization which cross-reacts with peripheral nerves due to molecular mimicry. This reaction affects the myelin or the axon of peripheral nerves, resulting in neuron damage. Diagnosis is mainly clinical as there are few biomarkers available for the majority of variants, the most notable of which are antibodies directed against gangliosides. Clinical diagnostic criteria include progressive, relatively symmetric, weakness, and areflexia. Additional testing with EMG and nerve conduction studies can be helpful but are not required to diagnose. The Brighton criteria are a set of guidelines aimed at facilitating the diagnosis of GBS by incorporating the aforementioned clinical and electrodiagnostic data as well as laboratory values [17]. Electrodiagnostic criteria for the diagnosis of definitive GBS are defined by NCS showing the presence of two abnormal motor nerves as well as a sural-sparing pattern of sensory abnormality [18]. Both clinical and electrodiagnostic criteria were present in our patient.

Most notably in 1976, a spike in cases of GBS was seen associated with vaccination for the H1N1 influenza A virus with roughly one in 100,000 people developing GBS [17]. Emerging literature is starting to show episodes of GBS associated with several different COVID-19 vaccinations. In a 2022 British study, 198 cases of GBS were documented within six weeks of receiving the first dose of a COVID-19 vaccine. Of note, AstraZeneca’s COVID-19 vaccine accounted for 176 of these cases, and Moderna’s vaccine for one. There was no increased risk of GBS shown after the second dose of any vaccine [12]. Additionally, the United States Food and Drug Administration published a press release in 2021 regarding the increased risk of GBS associated with the Johnson & Johnson COVID-19 vaccine [19]. Furthermore, in a 2021 Israeli study monitoring 701 patients with a prior history of GBS who received COVID-19 vaccination, only one developed signs and symptoms of a recurrence [20]. Pathognomonic laboratory findings include a CSF analysis showing albuminocytologic dissociation or an increase in CSF protein without an increase in cells. In a 2019 study by Bourque et al., the average CSF protein concentration in patients with GBS was 123 mg/dL, with increased protein levels associated with lumbar punctures taken at least seven days from symptom onset (normal range: <45 mg/dL) [21]. Additionally, increased values of CSF protein are seen with demyelinating subtypes of GBS [22]. The presence of pleocytosis of greater than 50 WBC/mm^3^ often suggests alternative diagnoses; however, there have been documented cases of GBS where pleocytosis is present [23,24]. Classical symptoms of GBS include progressive bilateral weakness, with decreased tendon reflexes [17]. Patients with GBS often present with symptoms several days to weeks after insult, ranging from mild weakness to complete paralysis although there are no criteria regarding a timeline for diagnosis [25].

Our patient presented with weakness, decreased lower extremity deep tendon reflexes (DTR) and ataxia following recent COVID-19 vaccination, insect exposure, and newly diagnosed syphilis infection, with no prior history of syphilis or syphilis treatment. Additionally, CSF findings demonstrated a reactive VDRL, markedly elevated protein, pleocytosis, and elevated glucose. The CSF findings of elevated protein found in NS, combined with the pleocytosis not typically seen with GBS, suggested a mixed pathologic picture. Additionally, recent COVID-19 vaccination was a potentially contributing factor which provided a diagnostic conundrum [12,14,19,21,23].

While initial improvement with IVIG suggested a sole diagnosis of GBS, such improvement has been documented before in a case report detailing NS treated with IVIG prior to definitive treatment with PCN G [11]. Typically, serum RPR will rise in early syphilis infection and peak during the secondary stage with a decline over time, even in the absence of treatment [26]. Our patient’s high serum RPR and positive CSF VDRL combined with his skin rash were consistent with disseminated spirochetemia and early NS which implied that this infection was relatively new. The presence of symptoms of late NS, especially the decreased reflexes resembling tabes dorsalis, would be abnormal for this timeline. Additionally, his MRI of the brain and spine was negative for acute pathology or signs of myelitis. His inpatient EMG of the distal extremities, however, demonstrated evidence of neuropathy consistent with GBS [27,28]. While it is true that diabetic patients often suffer from neuropathy, our patient's lack of previous neuropathy as well as presence of weakness which is not commonly seen in diabetic neuropathy further supports the diagnosis of GBS [29].

We postulate that our patient suffered from both confirmed secondary syphilis with early NS and concomitant GBS, possibly triggered by the syphilis infection and/or recent COVID-19 vaccination. The presence of peripheral neuropathy, not normally seen in early NS, alongside a positive CSF VDRL, and negative Lyme disease test, supports this hypothesis. Further evidence to support this was demonstrated by early improvement with IVIG. Interestingly, even dating back to an article by Garvey et al. (1940) on GBS where he encountered patients presenting with syphilis and GBS, he suggested that the patients’ symptoms may have been precipitated by an infectious agent after having ruled out other causes [3]. It should be noted for the sake of completion that while there exists the possibility of false-positive treponemal testing, especially in patients with autoimmune or collagen disorders, we do not believe this was a false-positive result especially in light of his symptoms, serology, and negative antinuclear antibody (ANA) and double-stranded DNA (DS-DNA) tests [30,31]. More research and documented cases of syphilis presenting with GBS are clearly required to better understand this and if causality can be demonstrated.

## Conclusions

To our knowledge, this is the first case report documenting three simultaneous processes, infectious, immune mediated, and vaccine related, contributing to one overarching presentation. Syphilis is often referred to as the great mimicker, and this case is no different. A thorough history and physical and expedient laboratory work made this diagnosis of neurosyphilis possible; however, cautious clinical acumen led to the diagnosis of simultaneous neurosyphilis and Guillain-Barre syndrome. The novel COVID-19 vaccination may have also factored into this clinical scenario as well. We suggest more research is necessary to study the relationship of these coexisting pathologies.
